# The Use of Selective Laser Melting to Increase the Performance of AlSi_9_Cu_3_Fe Alloy

**DOI:** 10.3390/ma11101918

**Published:** 2018-10-09

**Authors:** Michaela Fousova, Drahomir Dvorsky, Marek Vronka, Dalibor Vojtech, Pavel Lejcek

**Affiliations:** 1 Department of Metals and Corrosion Engineering, University of Chemistry and Technology Prague, Technicka 5, 166 28 Prague 6, Czech Republic; dvorskyd@vscht.cz (D.D.); vojtechd@vscht.cz (D.V.); 2 Institute of Physics, Academy of Sciences of the Czech Republic (AS CR), Na Slovance 1999/2, 182 21 Prague 8, Czech Republic; vronka@fzu.cz (M.V.); lejcekp@fzu.cz (P.L.)

**Keywords:** aluminum alloy, AlSi_9_Cu_3_(Fe), selective laser melting, additive manufacturing

## Abstract

For the first time, the comprehensive characterization of the additively manufactured AlSi_9_Cu_3_Fe alloy is reported in this paper. Conventionally, the AlSi_9_Cu_3_(Fe) alloy is prepared by high-pressure die casting (HPDC), but this technology largely does not offer such opportunities as additive manufacturing (AM) does, especially in the design of new lightweight parts. In the present paper, testing samples were prepared by selective laser melting (SLM), one of the AM technologies, and characterized in terms of their microstructure (by means of light microscopy, scanning electron microscopy and transmission electron microscopy in combination with analytical techniques for evaluation of chemical and phase composition) and mechanical properties (static tension, compression, and hardness). All the characteristics were compared with the HPDC reference material. Our study showed an excellent improvement both in strength (374 ± 11 MPa compared to 257 ± 17 MPa) and plasticity (1.9 ± 0.2% compared to 1.2 ± 0.5%) of the material thanks to its very fine and distinctive microstructure.

## 1. Introduction

In the last decades, the industry looks for new, sophisticated technologies with the intention of making technological progress, but also reducing production costs. As the environmental protection aspect is very strong in this age, it also represents one of the driving forces of the research and development. Especially in the aerospace and automotive industries, all these requirements have led to the development of lightweight materials and structures. With reduced weight, not only material costs are saved, but it also lowers the weight of the resultant means of transport associated with lower fuel consumption, as well as lower emissions of harmful exhaust gases (especially CO_2_) [1]. However, traditional manufacturing processes, such as casting, forging, extrusion, or powder metallurgy, are not able to satisfy the current trend in manufacture, which aims at new customized products of high quality, acceptable cost, repeatability and reliability, and their quick delivery to customers. Conversely, they require specific tooling, consist of multiple steps and often have to be followed by post-fabrication machining, which increases both costs and production time [2]. A new technological approach is thus needed.

Aluminum and its alloys find their application thanks to their excellent strength to weight ratio and corrosion resistance (compared to steels). The most wide-spread aluminum alloys are based on the binary Al-Si system, which provides great castability, and thus an easy production way. Elements like Mg or Cu are often added to enhance mechanical properties after an appropriate heat treatment [1]. AlSi_9_Cu_3_(Fe) alloy is used especially in the automotive industry. Thanks to a relatively high content of copper, it exhibits higher mechanical strength (when 3 wt.% of Si in AlSi12 are substituted by copper, the contribution to tensile strength is 90 MPa [3]), but decreased corrosion resistance. It is usually processed by pressure die casting and is easy to machine. It has a low tendency towards surface defects and internal voids during solidification. Particularly, it is used for the production of thin-walled products subjected to dynamic loading, cylinder heads, and other parts of engines or various machines [4]. Mechanical properties can be significantly influenced by the control of microstructural parameters, such as the distance between dendritic arms, grain size, shape, and distribution of the eutectics, volume fraction, size, and morphology of intermetallic phases [5].

High-pressure die casting (HPDC) enables high production volumes of parts showing high surface quality. Compared to gravity casting, even more complex shapes are possible to be produced, but still, the current demands for porous structures or very small dimensions are hardly attainable. Additionally, the HPDC process is limited by the formation of defects, such as oxide films, shrinkage cavities, air porosity, etc., which cannot be eliminated. Such defects then weaken the castings structurally and exclude them for use in the field of safety applications [5].

The opportunity to satisfy industrial producers and their customers, and avoid limitations of conventional technologies is provided by additive manufacturing (AM), popularly called 3D printing. AM covers all computer-controlled processes that create three-dimensional (3D) parts by sequentially joining thin layers of materials. This unique feature makes it possible to produce complex parts directly in the desired form, without the need for expensive tools or molds, the use of which is necessary in conventional subtraction technologies. The additive approach also brings minimal material losses. An added value is a customized production. Components can be tailored according to the specific requirements of each customer. For these reasons, 3D printing is now widely considered for the production of high-performance components for aerospace, medical, power, and automotive applications [6,7,8].

In the AM of metals, the most available technologies are selective laser melting (SLM) and electron beam melting (EBM). They belong into the group of so-called powder-bed AM technologies. They are based on the selective melting of an input powder material that is deposited onto the working plate in successive steps so that thin layers are formed. While SLM uses a laser beam to melt the powder, a focused beam of electrons serves as the heat source in EBM [9,10]. 

Currently, 3D printing is applied for a wide range of metallic materials, including steels, Ti and its alloys, Ni or Co superalloys, and copper [11]. Additionally, some aluminum powders have been successfully processed, with AlSi10Mg alloy being one of the most studied [12]. Other studies have focused on different Al-Si alloys, e.g., A356 [13], A357 [14], AlSi12(Mg) [15,16], Al-20Si [17], and Al-50Si [18]. Wrought alloys of 2xxx, 6xxx, and 7xxx classes, usually heat-treatable (EN 7075 [19], Al-Mg-Sc-Zr [20], AA-2024 [21]), have been processed as well. An interesting paper was also published on the topic of SLM applied for processing of an Al–Fe–V–Si alloy which gets very close to titanium in its strength. While a complicated preparation and high costs had hindered the development of such alloys, SLM brought promising options [22]. Similarly, SLM enabled the preparation of a high-strength thermally stable Al85Nd8Ni5Co2 alloy with a composite-like microstructure containing submicrometer-sized intermetallic phases dispersed in the aluminum matrix [23]. While most of the materials for AM are based on conventional compositions, there are already some works tailoring materials specifically for AM [24,25].

Surprisingly, to the best of our knowledge, no references to the AM of the AlSi_9_Cu_3_(Fe) alloy have appeared in the literature yet. As the high amount of copper in combination with high cooling rates during the AM process could yield in interesting properties (e.g., absence of intermetallic phases enhancing plasticity, high oversaturation of solid solution, and promoted strengthening), the aim of our study was to prepare this alloy by means of SLM and characterize its microstructure and mechanical performance. Therefore, in the present paper, we bring a comprehensive study in which the SLM AlSi_9_Cu_3_Fe alloy is compared to the same alloy, but prepared by conventional HPDC.

## 2. Materials and Methods

**Figure 1:**
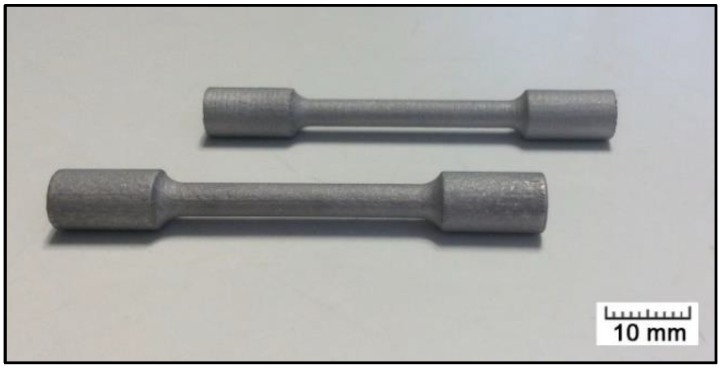
Tensile test samples prepared by SLM.

The tested material was the AlSi_9_Cu_3_Fe alloy of a specific composition given in Table 1. A gas-atomized powder of the alloy (LPW, mass median diameter = 40 μm) was used for the purpose of additive manufacture by the selective laser melting technology (SLM). SLM Solution 280HL machine equipped with 400 W YLR-Faser-Laser was used. The process parameters applied for the sample production are listed in Table 2. The laser melting was carried out under a protective argon atmosphere to prevent oxidation. Dog-bone-shaped samples intended for tensile testing (Figure 1) were prepared directly, with their longitudinal axes parallel to the building direction. Sand-blasting followed the additive manufacture to remove powder particles attached to the final sample surface.

For a comparison between SLM and conventional manufacture, castings produced by HPDC were provided by a commercial supplier. Samples for determination of mechanical properties and structural analysis were cut out of the castings.

For the microstructure observation of the studied alloy, metallographic sections were prepared in a standard metallographic way. For the SLM samples, both transversal and longitudinal sections were prepared because of additive manufacture directionality. Therefore, the longitudinal sections represented the microstructure in the building direction, while the transversal ones in the direction perpendicular to the building direction. First, porosity was evaluated using unetched samples. The average porosity was determined by an image analysis (ImageJ software) of about 30 images captured by a light metallographic microscope (OLYMPUS PME3) across the entire longitudinal section. To reveal the microstructure, the samples were etched in 0.5% HF. Microstructures were studied by light microscope and also by a TESCAN VEGA-3 LMU scanning electron microscope (SEM) equipped with an EBSD (electron backscatter diffraction; Oxford Instruments, Aztec) analyzer. The EBSD analysis was performed with a step of 0.3 μm. Data were processed by Channel 5 software. Transmission electron microscopy (TEM) was used to observe nano-sized microstructural features. TEM samples were prepared perpendicular to the building direction of SLM. 1 mm thick plates were cut and reduced by grinding to a thickness of 100 μm. Subsequently, disks of 3 mm in diameter were punched. The final thickness in the central part of the disks was achieved by double jet electropolishing in a 30% solution of HNO_3_ in methyl alcohol at 253 K. The conventional TEM observations were carried out by Fei Tecnai F20 field emission gun transmission electron microscope operated at 200 kV equipped with EDS detector. EDS analysis was performed in the STEM mode with a step size of 1 nm. Phase composition was studied locally by SAED (selected area electron diffraction) and globally by X-ray diffraction using PANalytical X’Pert PRO diffractometer equipped with Cu anode.

For a comparison between the SLM and the cast alloy, mechanical properties were tested. Uniaxial tensile tests were done with 3 samples using a universal testing machine LabTest 5.250SP1-VM. For compressive tests, cylinders of 8 mm in diameter and 12 mm in height (2:3 ratio) were used. Both tensile and compressive tests were accomplished at room temperature with a strain rate of 0.001 s^−1^. Hardness measurement was carried out on a Future-Tech FM-700 hardness tester and the Vickers hardness HV1 was determined. Fracture surfaces were studied by scanning electron microscopy.

## 3. Results and Discussion

### 3.1. Microstructure

#### 3.1.1. Hierarchical Microstructure of the Additively Manufactured AlSi_9_Cu_3_Fe Alloy

**Figure 2:**
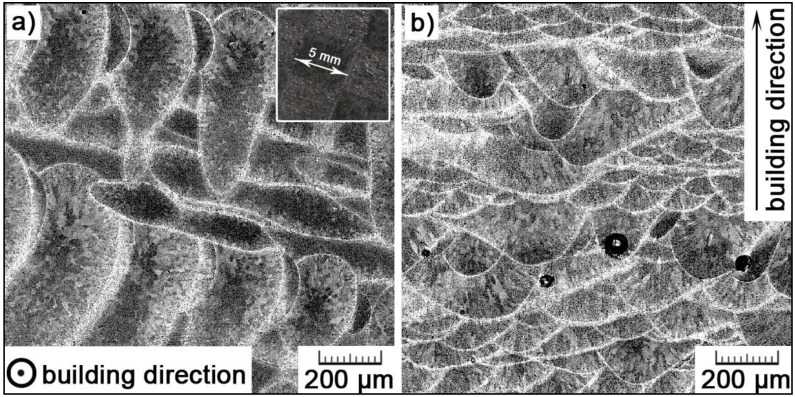
Macrostructure of the SLM AlSi_9_Cu_3_Fe alloy in (**a**) transversal and (**b**) longitudinal section (image inserted in (**a**) represents a macroscopic view of the chessboard scanning strategy).

As with other metallic materials prepared by SLM, the microstructure of the AlSi_9_Cu_3_Fe alloy also shows hierarchical heterogeneity, with length scales spanning nearly six orders of magnitude [26]. In the first magnification range, the additively manufactured AlSi_9_Cu_3_Fe alloy displays very characteristic macrostructure, which is related to the principle of its manufacture—the successive melting of the powder material by the laser beam. The laser beam was scanned across each powder layer according to the selected scanning strategy. By selective irradiation of the powder, melt pools were formed transiently [27]. After solidification, these melt pools are visible in the macrostructure of the processed alloy. The characteristic macrostructure of the studied AlSi_9_Cu_3_Fe alloy is shown in Figure 2. In the transversal section (Figure 2a), a top view of a layer is displayed. Here, several oval zones elongated in the same direction can be seen. The direction of laser scanning and the thickness of a laser track can be estimated. However, we can distinguish two types of elongated zones that overlap perpendicularly as the scanning direction changed with each layer by 90°. In our work, a ‘chessboard’ strategy was applied, meaning that every layer was divided into square boxes representing a chess board. The area of every box was scanned in one direction, with a firmly set hatching distance in between adjacent laser tracks. The scanning direction changed alternately for neighbor boxes. In the next layer, the directions were switched. In Figure 2b representing the longitudinal section, consistent with the building direction, solidified melt pools can be observed directly. Their depth overpasses the layer thickness by a factor of about 1.5–4, which ensures a proper interconnection between layers.

**Figure 3:**
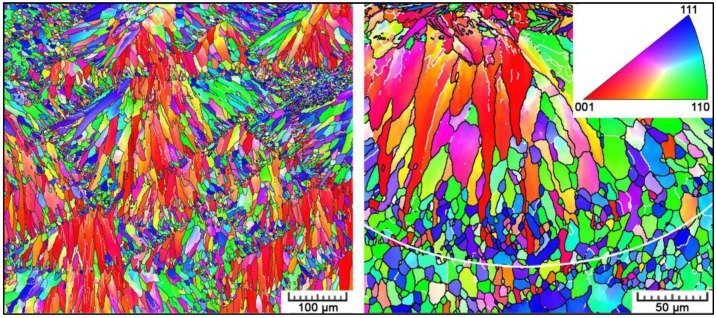
IPF maps constructed for fcc α-Al phase with the orientation triangle corresponding to the building direction (high-angle boundaries in black, low-angle boundaries in white). White line indicates the melt pool boundary.

Thanks to a slight contrast provided by the bright-field light microscopy, in Figure 2, individual grains can be estimated within the melt pools. However, much better information is provided by EBSD maps shown in Figure 3. Individual grains within a melt pool, as well as on its boundaries, can be clearly distinguished. The grain growth is directly influenced by temperature distribution within melt pools. At melt pools boundaries, where the heat is quickly conducted away by the already solidified and chilled material, fine equiaxed grains are formed by the heterogeneous nucleation occurring in front of the liquid-solid interface. The slowest heat dissipation occurs in the center of a melt pool where the highest temperature is kept for the longest period of time. Therefore, larger grains can be observed in melt pool interiors. As a melt pool is surrounded by already solidified material, the heat flows away radially. Therefore, within a melt pool, grains elongate in the direction of the thermal gradient, converging from boundaries of the melt pool to its center [28,29]. At the top surface, fine equiaxed grains are also formed as the cooling rate was reported (e.g., in References [28,29]) to reach ~10^6^ K/s here (compared to ~10^3^ K/s deeper in the melt pool).

Due to a direct heat flow in the steep temperature gradient between the liquid melt pool and the previously consolidated layer, in alloys, the liquid may become undercooled due to the solute redistribution. That destabilizes the solidification front and generates a transition from a planar solidification mode to a cellular or dendritic mode. This transition occurs when the thermal gradient in the liquid phase at the solidification front becomes lower than the critical gradient, which is a function of the overall solute concentration, the solute diffusion coefficient in the liquid phase, the solute partition coefficient *k*, the solidification growth rate *R,* and the gradient of the equilibrium melting point. In multi-element alloys, the critical gradient derived for binary systems can be influenced by other alloying elements by changing the partitioning coefficients. The addition of an extra alloying element with a large partition coefficient increases the critical gradient, destabilizes the solidification front and promotes cellular solidification. The kinetic conditions favor the solidification of a low melting phase in the first step by ejecting high melting solute(s) at the solid-liquid interface. The low-melting phase thus occupies the core of the cells and the high-melting phase forms cell boundaries [30]. However, Prasanth and Eckert [31] suggested that apart from thermodynamic and kinetic considerations, also the surface tension aspect should be considered in rapidly solidified SLM specimens due to the SLM process specific characteristics. A melt pool is quite narrow, with at least one side surrounded by solid material, one or two sides surrounded with powder particles, and the top of the pool in contact with a protective gas. Such melting environments lead to a non-uniform heating and generation of thermo-capillary convection caused by strong surface tension effects. The cellular microstructure is thus a result of a surface tension driven instability termed the Benard Marangoni surface instability.

**Figure 4:**
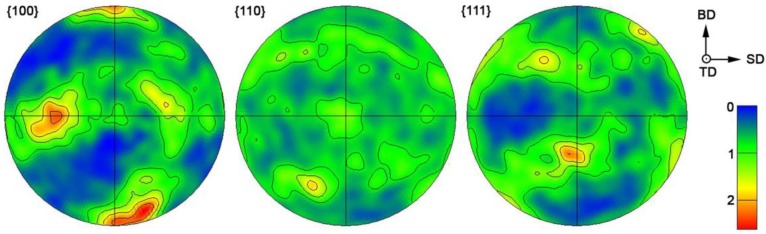
Pole figures constructed for fcc α-Al phase. The orientation of the specimen coordinate system and the relative intensity of the diffraction peaks are shown.

When the cellular solidification mode is active, cubic materials are known to preferentially grow the cells along the 〈1 0 0〉 crystal direction. Therefore, not only a morphological texture is created, but also a crystallographic texture. Small equiaxed grains at melt pool boundaries are newly nucleated grains among which a competitive growth occurred. However, at places where the 〈1 0 0〉 direction of the substrate (previously solidified material) is along the heat flow direction, grains grow epitaxially. As only a few orientations can grow further towards the melt pool center, we can observe large elongated grains in melt pool interiors [29]. Based on the orientation triangle in Figure 3, one can see that elongated grains have their 〈1 0 0〉 orientation parallel to the building direction, and in small equiaxed or slightly prolonged grains 〈1 1 0〉 and 〈1 1 1〉 orientations prevail. Pole figures in Figure 4 testify to the 〈1 0 0〉 cube texture along the building direction.

**Figure 5:**
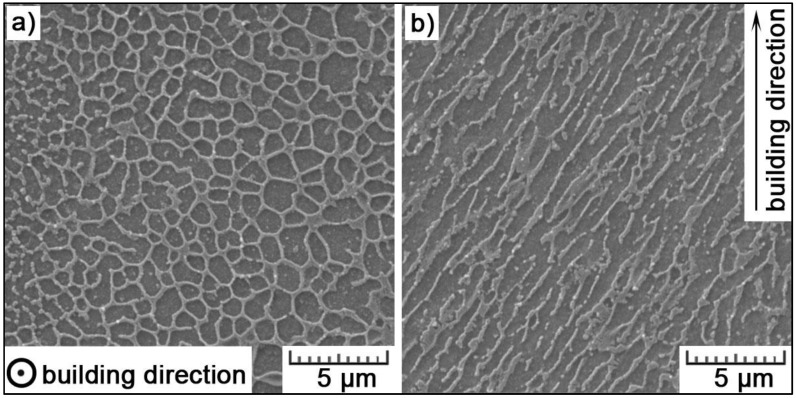
Fine cellular substructure in (**a**) transversal and (**b**) longitudinal section.

**Figure 6:**
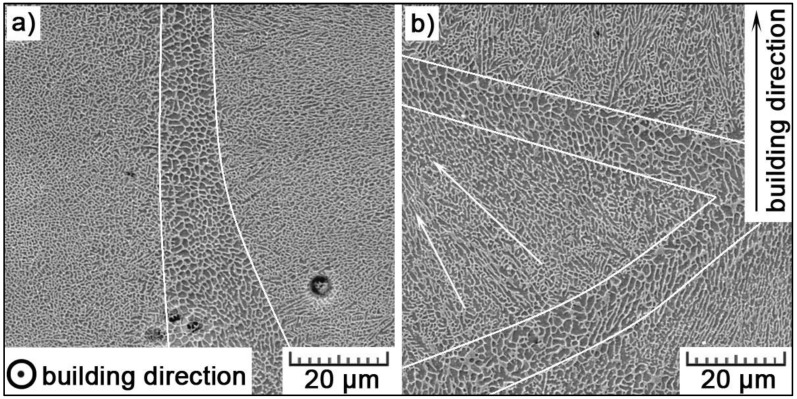
Melt pool boundaries in (**a**) transversal and (**b**) longitudinal section (arrows show the directionality of cells towards the melt pool center).

When lowering the length scale further, very fine cellular-dendritic substructure can be observed within the grains (Figure 5). The solidification mode is mainly cellular, but occasionally, some side branches can be seen. It is a result of rapid cooling. Due to a very short persistence of a laser beam focused on a specific spot of a powder layer, the powder material is melted instantaneously, with high temperatures reached, and solidified the very next moment. Temperature gradients between the melt, and its surrounding (up to 10^5^ K/m) and the cooling rates (10^6^–10^8^ K/s) are very high [32]. The formation of dendrites with multiple arms is impossible at such rates. Therefore, very fine cells are formed. They elongate in the direction of the highest temperature gradient (the elongation in one direction is clear from the longitudinal section in Figure 5b). Their size reaches 1.0 ± 0.1 μm in diameter and 3.7 ± 0.8 μm in length. In Figure 6b, it is visible that the cells orientate towards the center of the melt pools. The cells are formed by α-Al solid solution and are surrounded by the network of eutectic Si. The eutectics fraction was assessed by image analysis and represents 29 ± 5 vol.%. As long as the cells have the same crystallographic orientation, they form one grain.

SEM images in Figure 6 show the boundaries of ‘melt pools’, about 10–15 μm thick. Here, the cellular substructure is coarser, what can be explained by the Gauss distribution of laser energy. The undercooling changes over the melt track; it reaches the maximum at the centerline, then gradually decreases and goes to the minimum at the boundary of the melt track [33]. From this point of view, the cellular size attains the minimum at the center of the melt track, while it reaches the maximum value on its boundary.

**Figure 7:**
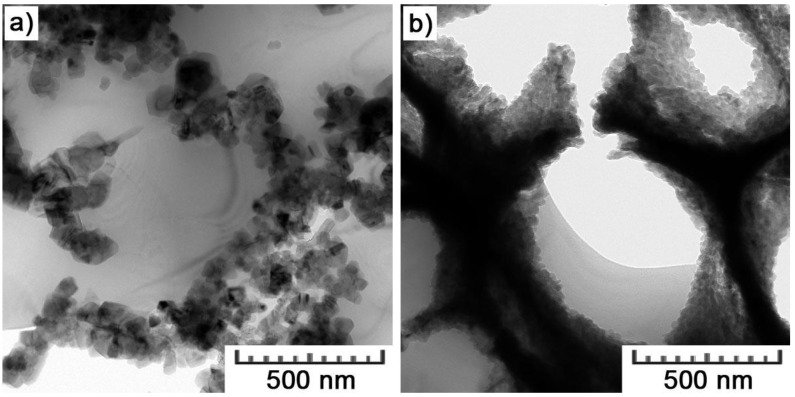
TEM bright field images obtained in the area of (**a**) a melt pool boundary and (**b**) a melt pool interior.

The greatest details are provided by TEM images in Figure 7. It can already be distinguished that the cell boundaries are formed by a network of silicon particles. These particles are cubic with an edge length of 30–70 nm. A significantly higher density of Si particles can be observed within the interior of melt pools (Figure 7b). Such a difference can be explained by a different thermal history. With higher undercooling inside melt pools, more numerous, and finer particles are formed [34].

#### 3.1.2. Microstructure of the Conventionally Prepared HPDC AlSi_9_Cu_3_Fe Alloy

**Figure 8:**
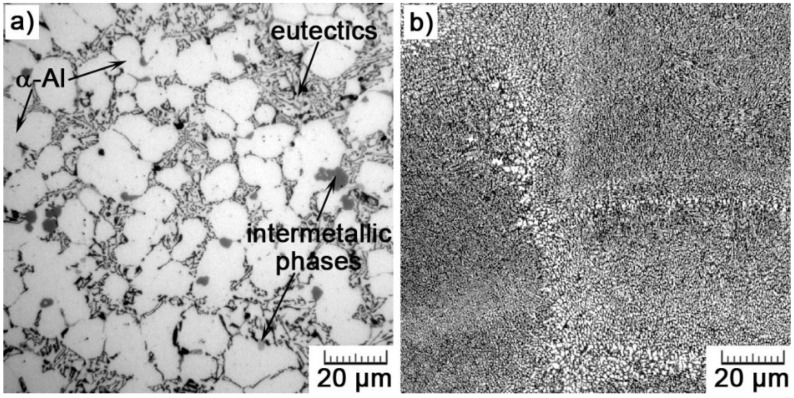
Comparison between (**a**) as-cast (HPDC) and (**b**) SLM microstructures.

The comparison of optical micrographs in Figure 8 clearly displays the difference between the additively manufactured and conventionally cast alloy. The as-cast microstructure consists of α-Al dendrites, α-Al+Si eutectics and intermetallic phases (many phases can be possibly formed; binary (e.g., θ-Al_2_Cu, Mg_2_Si), ternary (e.g., β-Al_5_FeSi, Al_9_Fe_2_Si, Al_2_CuMg, Al_15_Mn_3_Si_2_), or quaternary (e.g., α-Al_15_(MnFe)_3_Si_2_, A_l5_Mg_8_Si_6_Cu_2_) [35,36,37]). The eutectics is of a lamellar type with platelets of Si. The volume fraction of the eutectics was determined to be 51 ± 5%.

At first sight, the as-cast microstructure is much coarser than that in the AM material. The equivalent diameter of 18.4 ± 4.5 μm for dendritic branches overpasses the size of cells (1.0 ± 0.1 μm in the cross-section) by a factor of 18. That can be explained by the difference in cooling rate during both manufacturing processes. While cooling rates of HPDC ranges 10^1^–10^2^ K/s [38], they can reach up to 10^8^ K/s during SLM [32]. Additionally, the size of Si plates is significantly larger (average length of 22 ± 6 μm and thickness of 0.1–2.0 μm) than nano-sized cubes of Si forming the cellular network (Figure 7). Generally, such plate-like morphology of Si is not good for mechanical properties, because Si platelets are hard and brittle, and so reduce ductility and tensile strength. Therefore, there are various approaches used to affect the morphology of Si in an appropriate manner [39].

### 3.2. Chemical and Phase Composition

#### 3.2.1. The Additively Manufactured AlSi_9_Cu_3_Fe Alloy

Due to the very fine microstructure of the AM alloy, the EDS analysis was carried out in the STEM regime. It revealed that the oversaturated solid solution of α-Al contains 2.2 ± 0.3 wt.% of Si and 4.6 ± 2.7 wt.% of Cu. At high solidification velocities (here up to 10^8^ K/s), the deviation of liquidus and solidus lines from equilibrium values occurs, leading to a solute trapping effect. A transition from diffusion-controlled to diffusion-less solidification is predicted as the interface speed exceeds the maximum speed with which solute atoms can diffuse across the interface and are thus pinned down in the product phase [40].

**Figure 9:**
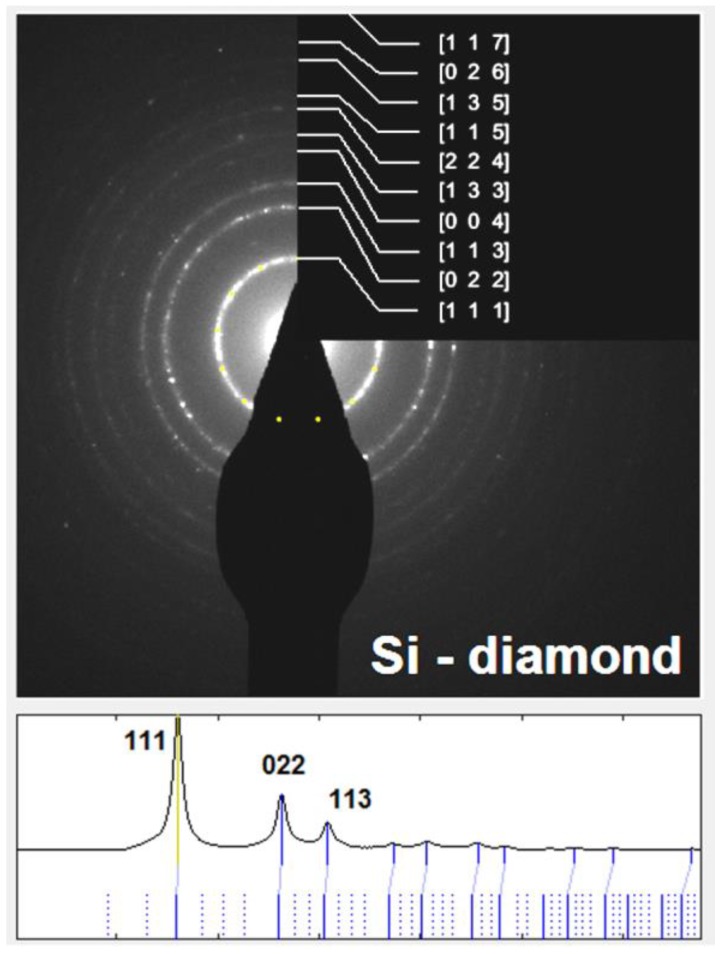
Diffraction pattern and its evaluation.

**Figure 10:**
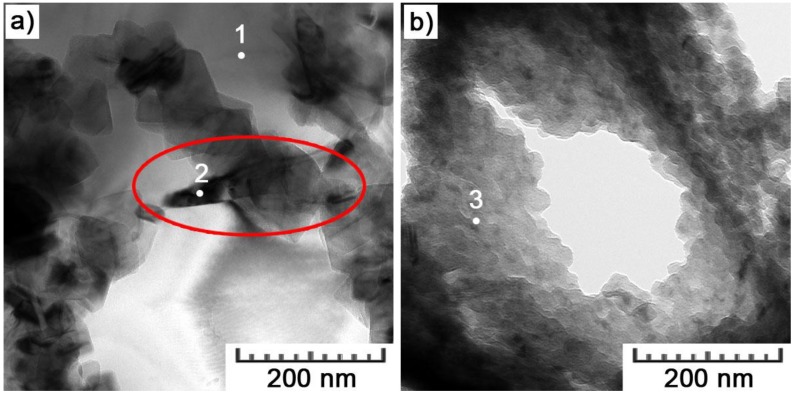
TEM bright field images showing (**a**) a CuAl_2_ particle and (**b**) area of Fe detection in the intercellular network (numbered points represent locations of point EDS analyses).

In the area of the Si network, the EDS analysis was already distorted by the surroundings. Nevertheless, it showed almost pure Si, which was confirmed by diffraction (Figure 9). Diffraction rings perfectly matched crystalline planes of Si with the diamond crystalline structure. Another type of particle (Figure 10a) was detected in the area of the intercellular network as well. It was shown to be composed only of Al and ~30 at.% of Cu (Table 3, 53.7 weight % corresponds to ~30 atomic %). It thus corresponds to the CuAl_2_ phase. X-ray diffraction determined 2 vol.% of this phase in the bulk material.

Iron was detected in the areas of fine Si particles in the interior of melt pools (Figure 10b). As no specific particles were distinguished in the cloud of Si particles, it is not clear what type of phase Fe forms. Along with 4.8 ± 0.8 wt.% of Fe, a small amount of oxygen was also detected (2.8 ± 0.2 wt.%). Data from point EDS analyses, complementing Figure 10, are given in Table 3.

#### 3.2.2. The Conventionally Manufactured AlSi_9_Cu_3_Fe Alloy

**Figure 11:**
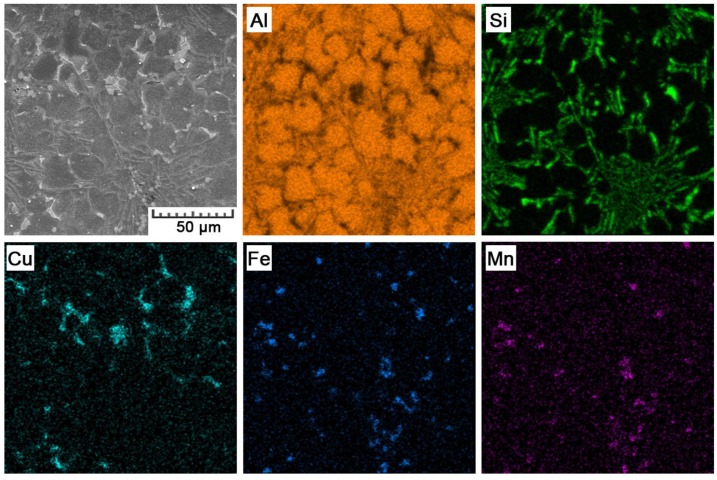
EDS maps showing the distribution of alloying elements in the HPDC AlSi_9_Cu_3_Fe alloy.

**Figure 12:**
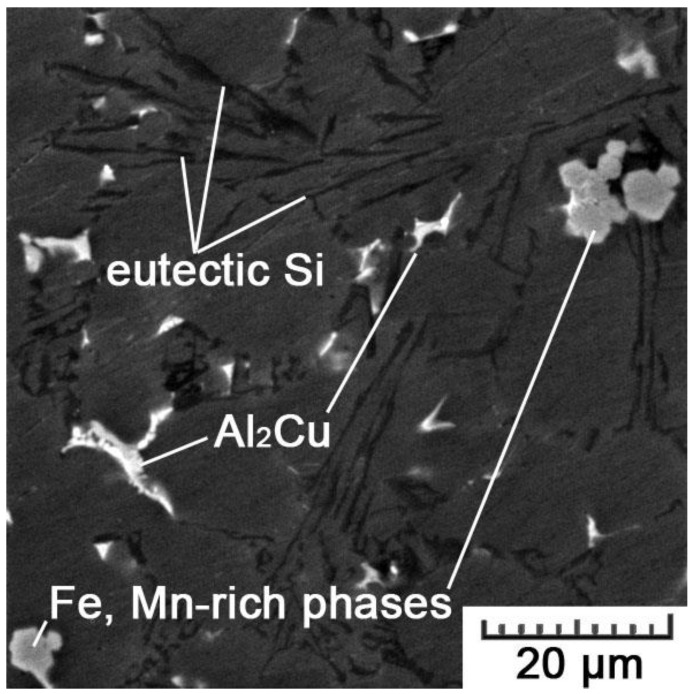
SEM image showing different types of phases in the HPDC AlSi_9_Cu_3_Fe alloy.

The distribution of constituting elements within the AlSi_9_Cu_3_Fe alloy prepared by HPDC is illustrated by EDS map in Figure 11. There are two main structural constituents—primary solid solution, and eutectics. The solid solution contains 1.9 ± 0.2 wt.% of Si and 0.9 ± 0.2 wt.% of Cu. Such super-saturation can be referred to the elevated cooling rate and pressure during the HPDC process. In eutectics, Si lamellae can be observed. Except these two main structural constituents, there are also two types of intermetallic phases. We were not able to determine their accurate composition due to the resolution limits of the EDS analysis. However, as EDS maps suggest, small pentagonal phases are formed mainly by Fe, Mn, and Cr, while larger irregular phases are predominantly formed by Al and Cu, but contain also small amounts of Mg, Ni, Sn, and Fe. Therefore, we can suggest that it concerns Al_15_(MnFe)_3_Si_2_ and Al_2_Cu phases, respectively. Their morphology and chemical compositions are shown in detail in Figure 12 and Table 4. In case of the Al_2_Cu phase, it is probably a ternary eutectic form (Al-Al_2_Cu-Si) that often precipitates on pre-existing Si-particles or Fe-phases, what explains the detection of other elements [36].

### 3.3. Mechanical Properties

**Figure 13:**
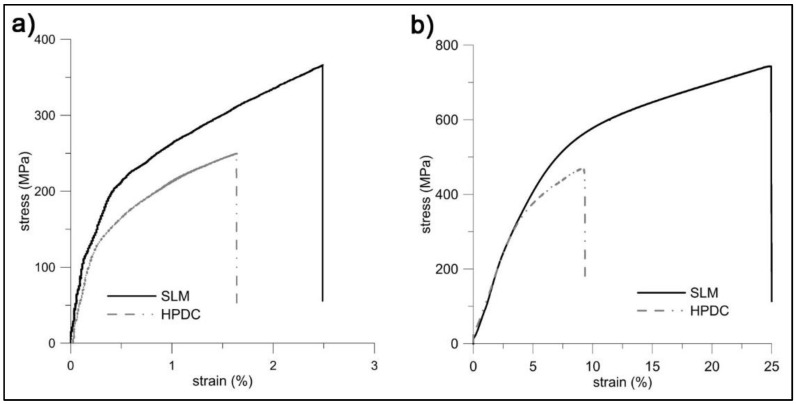
Representative stress-strain curves showing the comparison between the AlSi_9_Cu_3_Fe alloy prepared by SLM and conventional HPDC in mechanical properties: (**a**) Tension and (**b**) compression.

Figure 13 brings a comparison of stress-strain curves obtained for the SLM and HPDC AlSi_9_Cu_3_Fe alloy under tensile and compressive loading. Values of selected mechanical properties are then summarized in Table 5. The curves show significantly higher strength, but also plasticity of the SLM alloy, which is a unique asset. While it has been a longstanding challenge to overcome the strength-ductility trade-off that exists ubiquitously in pure metals and alloys, SLM has turned out to be capable of that [26]. Several papers has reported that the contradictory strength-plasticity relationship was overcome in some materials (e.g., stainless steel [26], AlSi10Mg alloy [41] or Ti6Al4V [42]).

Very fine cellular substructure (Figure 5) and the existence of the strongly oversaturated solid solution, both resulting from high cooling rates during the SLM process, yielded in stronger strengthening effect. As there was not such a significant difference in the content of solute atoms in the α-Al solid solution, it can be expected that the Hall-Petch strengthening is dominant compared to the contribution of solid solution strengthening. The Hall-Petch contribution can be determined according to the following equation:

(1) $$\Delta \sigma_{HP}=kd^{-1/2},$$

where *k* is the Hall-Petch coefficient and *d* is the grain size. The *k* value is not known for the investigated material, but for a simplified estimation, values reported in other research works can be used. In [43], coefficients for rapidly solidified aluminium alloys strengthened with particles were reported to range between 150 and 170 MPa μm^1/2^. When the average grain sizes determined for the additively manufactured and conventionally HPDC cast AlSi_9_Cu_3_Fe alloy (3.7 μm and 18.4 μm, respectively) are used in the Equation (1), the difference between the Hall-Petch contribution of both compared materials ranges 43–48 MPa. The real difference between TYS values reported in Table 5 is 46 MPa, which perfectly falls into this range. Therefore, we can deduce that the refinement brought by SLM plays the dominant role in increasing the strength of the AlSi_9_Cu_3_Fe alloy.

Moreover, the morphology and size of eutectic silicon are known to play a very important role, especially on material plasticity [33]. Therefore, small cubic particles of Si (Figure 7) are much more favorable than lamellar eutectic Si in the HPDC alloy (Figure 12). In conventionally cast material, acicular silicon acts as crack initiation sites and consequently results in low ductility. For that reason, different ways of the refinement of the eutectic microstructure of Al-Si alloys have been extensively investigated [44,45,46,47]. The refinement of the Si phase can be achieved by controlling the nucleation and growth of the eutectic grains. Usually, two different approaches are applied: elemental additions and rapid solidification. However, both have their drawbacks. Refining elements often evaporate, oxidize, and the modification can be thus hard to control. On the other hand, for most castings, rapid solidification is impossible to occur uniformly in the entire volume, and thus, such an approach is limited to very small and thin parts only [2]. Nevertheless, SLM circumvents these drawbacks. Due to the gradual melting and solidification, laser heats only a very small volume of a material during a short interaction time, so that high cooling rates (10^6^–10^8^ K/s [32]) are maintained throughout the entire volume of a fabricated part.

In Al-Si alloys containing Fe, the formation of β-Al_5_FeSi is also critical in terms of plasticity. This needle-like hard phase brings high stress concentrations and increases crack initiation. Its detrimental effect can be overcome by two approaches; rapid cooling or addition of a suitable neutralizing element [36]. In HPDC, manganese was added to convert the monoclinic β-phase to cubic α-phase Al_15_(MnFe)_3_Si_2_. In the case of SLM, there was no need of additional elements as high cooling rates prevented the formation of intermetallic phases by trapping them in the solid solution.

**Figure 14:**
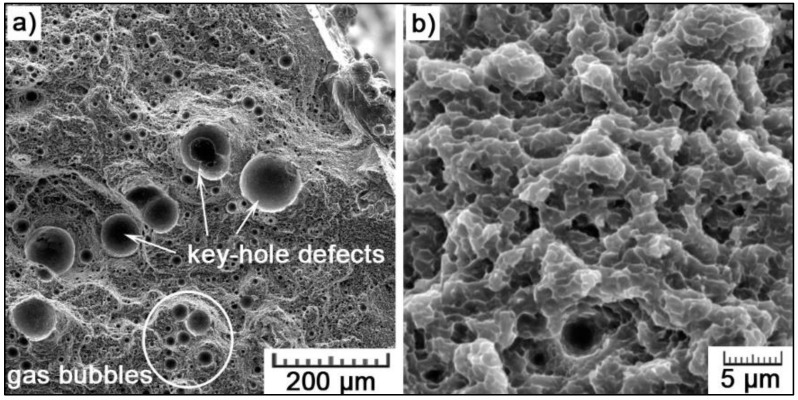
Fracture surface of the SLM AlSi_9_Cu_3_Fe alloy: (**a**) key-hole defects and gas bubbles; and (**b**) fracture morphology.

In tension, the increase in elongation is not so pronounced as the plasticity is limited due to the presence of defects. The total porosity of the samples was determined to represent 0.5 vol.%. Typical defects can be seen in Figure 14a in the fracture surface of an SLM sample subjected to the tensile test. It concerns two types of defects, both showing spherical shape. First, larger voids are so-called key-hole defects which are formed at the bottom of deep melt pools (as it can be seen in Figure 2b) due to the melt instability and evaporation of the metal by high-power laser beam. As the vapor cavity collapses, a void is formed. Secondly, smaller voids result from gas entrapment (probably oxygen) and its inability to escape from the melt during rapid solidification [29]. The fracture topography shows a ductile mode of fracture with extremely fine morphology (Figure 14b), which is linked to the Si enriched dendrite cell network. It is assumed that the samples fail along the cell network, where the fracture initiates due to the higher hardness caused by Si enrichment and reduced ductility compared to the Al-enriched cells [48].

## 4. Conclusions

In this study, the AlSi_9_Cu_3_Fe alloy, additively manufactured by SLM, was comprehensively characterized and compared with the same alloy, but prepared conventionally by HPDC. Compared to as-cast microstructure consisting of α-Al dendrites and lamellar Al-Si eutectics, SLM yields in hierarchically heterogeneous microstructure. Grains are arranged in melt pools representing material melted and solidified by single laser tracks in the direction of the highest temperature gradient. They exhibit very fine cellular substructure in which the cells of α-Al solid solution oversaturated in Si and Cu are separated by eutectic network formed by cubic particles of pure Si, here 30–70 nm in size. Altogether, the size of the cells lower than 1 μm, nanoscale cubic Si particles and oversaturation of the solid solution contribute to a significantly higher strength of the alloy. Microstructural features also favor the material plasticity. By elimination of internal defect, the plasticity could even be improved. Our study has thus shown that, compared to HPDC, SLM can desirably improve the performance of the AlSi_9_Cu_3_Fe alloy and extend its potential applications, which is also due to the possibility of SLM to produce complex lightweight structures.

## Figures and Tables

**Table 1:** Chemical composition (wt.%) of the AlSi_9_Cu_3_Fe alloy.

	Al	Si	Cu	Fe	Mg	Zn	Mn	Ti	Sn
SLM	bal.	8.9	3.1	1.2	0.3	0.01	/	/	/
HPDC	bal.	8.6	2.6	0.7	0.2	0.9	0.3	0.04	0.07
CSN EN ISO 42 4339 standard	bal.	8.0–11.0	2.0–3.5	max. 1.0	0.1–0.5	max. 1.2	0.1–0.5	max. 0.15	max. 0.1

**Table 2:** Parameters of the SLM process (P—power, v—scanning velocity, h—hatching distance, t—layer thickness).

P (W)	V (mm/s)	H (μm)	T (μm)	Scanning Strategy
400	1300	150	50	chess board

**Table 3:** Point EDS analyses (wt.%) (location of points in Figure 9).

Spectrum		Al	Si	Cu	Fe	O
1	solid solution	92.5	2.2	5.3		
2	CuAl_2_ phase	46.3		53.7		
3	area of Fe detection	18.6	71.1	3.1	4.3	2.8

**Table 4:** Chemical composition (at.%) of different types of phases in the HPDC AlSi_9_Cu_3_Fe alloy (Figure 12).

	Al_2_Cu	Al_15_(MnFe)_3_Si_2_
Al	69.5 ± 3.5	71.5 ± 2.7
Si	7.4 ± 3.0	12.2 ± 0.7
Cu	17.6 ± 2.8	1.1 ± 2.3
Fe	0.3 ± 0.1	9.2 ± 1.0
Mn		5.3 ± 0.7
Ni	0.6 ± 0.0	
Zn	0.7 ± 0.1	
Sn	0.1 ± 0.0	

**Table 5:** Measured mechanical properties of the SLM and HPDC AlSi_9_Cu_3_Fe alloy (TYS, tensile yield strength, UTS, ultimate tensile strength, A, elongation, CYS, compressive yield strength, UCS, ultimate compressive strength, and HV1, Vicker’s hardness with 1 kg load).

Production Technology	Tension			Compression		Hardness
	TYS (MPa)	UTS (MPa)	A (%)	CYS (MPa)	UCS (MPa)	HV1
SLM	219 ± 20	374 ± 11	1.9 ± 0.2	375 ± 30	752 ± 41	135.2 ± 4.8
HPDC	173 ± 14	257 ± 17	1.2 ± 0.5	342 ± 14	482 ± 58	108.1 ± 3.1
